# Assessment of Lemon Balm (*Melissa officinalis* L.) Hydrogels: Quality and Bioactivity in Skin Cells

**DOI:** 10.1155/2015/635975

**Published:** 2015-10-27

**Authors:** Kristina Ramanauskienė, Ada Stelmakiene, Daiva Majienė

**Affiliations:** ^1^Department of Clinical Pharmacy, Lithuanian University of Health Sciences, A. Mickevičiaus Street 9, Kaunas, Lithuania; ^2^Department of Drug Technology and Social Pharmacy, Lithuanian University of Health Sciences, A. Mickevičiaus Street 9, Kaunas, Lithuania; ^3^Neuroscience Institute, Lithuanian University of Health Sciences, Eivenių Street 4, Kaunas, Lithuania

## Abstract

The *aim* of the study was to design gels with lemon balm extract, assess their quality, and investigate the effect of rosmarinic acid on skin cells in normal conditions and under oxidative stress. *Methods*. The quantities of rosmarinic acid (RA) released from gels were evaluated by applying the HPLC technique. HaCaT cell viability was assessed by using the MTT method. ROS generation was measured using DCFH-DA dye. The *results* showed that the gelling material affected the release of RA content from gels. Lower and slower RA content release was determined in carbomer-based gels. After 6 hours of biopharmaceutical research *in vitro*, at least 4% of RA was released from the gel. The results of the biological studies on HaCaT cells demonstrated that, in the oxidative stress conditions, RA reduced intracellular ROS amounts to 28%; 0.25–0.5 mg/mL of RA increased cell viability by 10–24% and protected cells from the damage caused by H_2_O_2_. *Conclusions*. According to research results, it is appropriate to use a carbomer as the main gelling material, and its concentration should not exceed 1.0%. RA, depending on the concentration, reduces the amount of intracellular ROS and enhances cell viability in human keratinocytes in oxidative stress conditions.

## 1. Introduction

Lemon balm (*Melissa officinalis* L.) and its preparations have a mildly soothing, antiviral effect, improve the digestive tract, and relax intestinal spasms [1, 2]. Lemon balm is a natural source of rosmarinic acid (RA). RA is one of the main phenolic acids in the chemical composition of* Melissa officinalis* L., and it determines the pharmacological effect and the medical use of the plant [2–5]. Current studies have shown that lemon balm preparations have bacteriostatic, antimicrobial, and antiviral effects [3]. Currently, there are very comprehensive surveys that attempt to show the effect of lemon balm against herpes simplex virus [6–10]. Since lemon balm has antiviral effects, some authors state that they can be used against HIV-1 infection.* In vitro* studies have shown that lemon balm preparations inhibit HIV-1 reverse transcriptase [11]. There is evidence in scientific literature that lemon balm has antihistamine effects; thus it can be used externally by placing the cut grass on insect bites or other irritated areas [6, 12]. RA in lemon balm has demonstrated more active antioxidant activity compared to *α*-tocopherol [13]. Due to its antioxidant, anti-inflammatory, and immunomodulatory effects [3], RA, as part of the chemical composition of lemon balm, is effective in relieving symptoms of atopic dermatitis. The* in vivo* study conducted by Lee et al. (2008) showed that people with atopic dermatitis, who applied RA emulsion daily, had a decrease of erythema after 4–8 weeks and had the skin water loss decreased after 8 weeks [2]. It can be argued that, because of the antimicrobial, antiviral, anti-inflammatory, and antioxidant properties,* Melissa officinalis* L. can be used as a natural source of RA in the production of semisolid preparations characterized by antimicrobial and protective effects.

When modeling a semisolid preparation, it is highly important to choose appropriate base substances because they determine the physicochemical properties and therapeutic effects of the final product [14]. A suitable base ensures the stability of semisolid preparations during storage, good distribution on the skin, and an efficient release of a drug substance from the preparation [14]. Creams and hydrophilic gels are the most common topical preparations applied on the skin due to their positive sensory properties. The advantage that gel has over ointments or cream is that it is a more stable, nonslimy, and semisolid form of medication [15]. Due to its characteristic sensory properties (easily applicable on the skin, easily cleaned with water, and leaving no greasy membrane on skin, unlike other ointments), gel is suitable for the modeling of natural preparations that are usually applied several times a day for longer periods of time. Gel ensures easy and safe administration of such preparations at home by nonmedical persons [16]. Because of the aforementioned properties, it is important to produce gel containing lemon balm extract and to assess its quality using biopharmaceutical tests. Quality control testing of such topical semisolid dosage forms includes the identity, quantitative analysis, homogeneity, rheological properties, particle size determination, consistency, and medicinal substance release tests (Eur. Ph. 6.0; 01/2008:0132).* In vitro* release testing identifies the influence of a number of physicochemical parameters such as solubility of the drug substance in the carrier or the effect of particle size and viscosity of the dosage form on the release of the medicinal substance from the dosage form through a synthetic membrane into the acceptor medium [17, 18]. The results of scientific research revealed that rosmarinic acid is more easily released from emulsion bases than from ointments [19]. Because of the lack of data of biopharmaceutical scientific tests, it is important to assess the rate of RA release from modulated hydrophilic gels.

The aim of the study was to design pharmaceutical hydrophilic semisolid dosage forms with lemon balm extract, assess their quality, and investigate the protective effect of the main active substance (rosmarinic acid) on skin cells in normal conditions and under oxidative stress.

## 2. Materials and Methods

Experimental lemon balm hydrogel formulations were prepared according to the general technological principles of semisolid preparations [20]. The compositions of the formulated gels are presented in Table 1. These gels were developed to study the effect of individual gelling polymers on drug release behavior. Dry lemon balm extract was dissolved in water and dispersed in swelled gels under stirring. Carbomer gels (N1–N3) were prepared by neutralization with sodium hydroxide. Gels of methylcellulose (N7–N9) were prepared by overnight soaking of the polymer for complete hydration. Combination gels (N4–N6) were prepared by mixing polymer gels together [21].

### 2.1. Physicochemical and Biopharmaceutical Properties of Gels

The dynamic viscosity of gels was determined at room temperature using a Vibro Viscometer SV-10 (A & D Company, Ltd., Japan). The studied substance was placed into a special container for measurement. Subsequently, the container was fixed on the working surface of the device, and sensors were submerged into the studied substance. The rotation speed of a cylindrical spindle was 10.0 rev./min. The length of every measurement was 10 sec (*n* = 3).

The pH of semisolid preparations was analyzed using a pH meter HD 2105.1 (Delta OHM, Italy). A 5% solution was prepared to determine pH levels. The appropriate amount of the semisolid formula was topped with purified water and stirred for 30 minutes on an IKAMAG C-MAG HS7 magnetic stirrer (IKA-Werke GmbH & Co. KG, Staufen, Germany) at a temperature of 50°C. Then, the solution was cooled and filtered through a paper filter.

*In vitro* release experiments were performed using modified Franz-type diffusion cells. The semisolid sample (1.00 ± 0.02 g) was placed into the cell with a dialysis membrane. The dialysis membrane Cuprophan (Medicell International Ltd., UK) was made of natural cellulose. The area of the diffusion was 1.77 cm^2^. Purified water acted as the acceptor medium. The temperature of the acceptor medium was kept at 37 ± 0.2°C. The medium was stirred using a magnetic stirrer. Samples from the acceptor solution were taken at 1, 2, 4, and 6 h and were immediately replaced with the same volume of fresh acceptor solution.

Lemon balm extracts were analyzed by applying high-performance liquid chromatography. The capillary HPLC method was developed and validated. The amount of the RA was evaluated by applying the validated HPLC method, using the Agilent 1260 Infinity Capillary LC System (Agilent Technologies, USA) with an Agilent Diode Array Detector. The separation was performed in a C18 ACE (5 *μ*m) 150 × 0.5 id column. The mobile phase consisted of solvents, A (0.5% aqueous solution of acetic acid, *V*/*V*) and B (acetonitrile), using the following gradient elution: 23% of B at 0 min, 40% of B at 10 min, and 70% of B at 11–15 min; it subsequently returned to the initial conditions with 10-minute reequilibration, with the total run time of 25 minutes. The analysis was carried out at a flow rate of 10 *μ*L min^−1^, with the detection wavelength set at 330 nm.

### 2.2. Cell Lines and Cell Culture

Human keratinocyte cell line HaCaT was purchased from the Cell Lines Service GmbH (Germany). Cells of convenient concentration were seeded in culture flasks containing DMEM with 10% of fetal bovine serum, 100 U/mL penicillin, and 100 *μ*g/mL streptomycin. Cultures were then incubated at 37°C with 5% CO_2_ and saturated humidity; culture transfer was performed once a week, and the medium was renewed twice a week.

### 2.3. Measurement of Cell Viability

Twenty-four hours prior to testing, cells with the investigated preparations were transferred to 96-well plates at concentrations of 30,000 cells/well. The cells were incubated with (A) different concentrations of H_2_O_2_ and (B) different concentrations of RA and H_2_O_2_. After 24-hour treatment with the preparations, the DMEM medium was removed, and the cells were washed twice with 100 mL/well warm Phosphate Buffered Saline (PBS). After washing, 180 *μ*L/well PBS was added along with 20 *μ*L/well of 5 mg/mL MTT dye dissolved in PBS to each well. The cells were incubated with MTT at 37°C for 2 hours. Afterwards, the dye was removed, the intracellularly formed crystals were dissolved in DMSO (100 *μ*L/well), and the plate was kept in the dark for 15 minutes. The absorption was measured at 570 nm and 620 nm as reference with a microplate spectrophotometer (Sunrise, Tecan Group Ltd., Switzerland). The results were expressed as a fluorescence percentage in control cells.

### 2.4. Measurement of Intracellular ROS Generation

The production of ROS was assessed using the 2′,7′-dichlorofluorescein diacetate (DCFH-DA) as described in [22]. Twenty-four hours prior to treating cells with RA, they were transferred to 96-well plates at concentrations of 50,000 cells/well. After 24 hours, the medium was removed, and cells were washed with PBS. After washing, 200 *μ*L/well HBSS medium, supplemented with DCFH-DA (10 *μ*M), was added. The cells were incubated at 37°C for 30 min. After the incubation, the cells were washed twice with PBS and were subsequently treated with (A) different concentrations (0.05–0.5 mg) of RA or (B) 100 *μ*M of H_2_O_2_ and different concentrations of RA. The fluorescence of DCF was detected by a fluorometer at excitation and emission of 488 and 525 nm wavelengths, respectively. Data are presented as means of fluorescence intensity ± SE.

### 2.5. Statistical Analysis of the Results

All tests were done in triplicate. Mean values and standard deviations of the results were calculated by using Microsoft Office Excel 2010 and SigmaPlot version 12.0 (Systat Software Inc.) software. The significance of differences in test results was assessed by using Student's *t*-test. The differences were considered to be statistically significant when *p* < 0.05.

## 3. Results and Discussion

### 3.1. Evaluation of Physicochemical Properties of Hydrogels

For semisolid preparations that could be applied to the treatment of atopic dermatitis, natural medicinal plant material, dry lemon balm extract containing 80 mg/g of rosmarinic acid, was selected as the active substance. Gels produced with lemon balm extract were homogeneous and yellowish to brownish in color. According to the results (Table 2), gels that were produced with carbomer and the mixture of carbomer and methylcellulose [N1–N6] had higher pH values. Meanwhile, gels produced with the gelling material of methylcellulose [N7–N9] had weakly acidic pH: the values ranged from 5.84 to 6.32. It was observed that when the concentration of methylcellulose in these gels increased, the pH value of the gels was also increasing, and they became less acidic.

The results in Table 2 show that the selected gelling material affected the viscosity of the formulated gels. Gels [N1–N6] in which carbomer was used as the main gelling material had a higher viscosity, whereas methylcellulose-based gels [N7–N9] had the lowest viscosity. The latter semisolid systems were characterized by a liquid consistency. This shows that carbomer is a stronger gelling material compared to methylcellulose. The results confirmed the data published in scientific literature indicating that the concentration of gelling material has an influence on the viscosity of semisolid preparations; that is, increasing carbomer and methylcellulose concentrations in semisolid preparations increases their dynamic viscosity [15, 23]. Gels [N1–N3] in which carbomer was used as the main gelling material had the highest viscosity, whereas the lowest dynamic viscosity was found in methylcellulose-based gels [N7–N9]. Based on scientific literature, the pH value of dermatological preparations is an important quality indicator. In order to reach the maximum effect of these preparations, their pH should be close to that of the skin (5.4 to 5.9) [24]. In patients with atopic dermatitis, the pH of the skin may increase (6.0 to 6.5) [25]. The results of our study showed that the produced semisolid preparations are suitable for use on the skin: they are homogeneous and have suitable pH values and acceptable organoleptic properties.

### 3.2. Release of Rosmarinic Acid from Hydrogels

**Figure 1:**
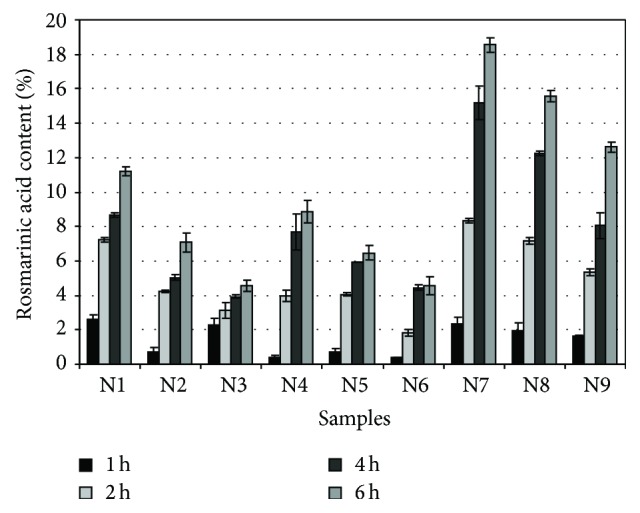
The kinetics of rosmarinic acid release from gels.

The results showed that the selected gelling material affected the release of RA content from semisolid systems (Figure 1). Lower and slower RA content release was more observed in carbomer and carbomer-methylcellulose-based combination gels than in the methylcellulose gels alone. In the evaluation of gels containing carbomer [N1–N3] as the main gelling material, the highest RA content was released after 2 hours, after which time a slowdown of the release was observed. Slower RA content release kinetics was observed in carbomer-methylcellulose-based gels [N4–N6]. The highest RA content was released after 4 hours, and then the release slowed down. The highest RA content was released from gels in which methylcellulose was used as the gelling material. Data of the statistical analysis showed a statistically significant difference (*p* < 0.05) between RA quantities released from methylcellulose-based hydrogels. Even though the highest released RA content was found in methylcellulose-based gels, carbomer due to its semisolid consistency is a more appropriate base for the insertion of liquid lemon balm extracts. Meanwhile, methylcellulose can be used for the production of gels in order to achieve a prolonged action of the medicinal preparation. Biopharmaceutical research proved that when the concentration of gelling materials in semisolid dosage forms is increasing, the released RA content is decreasing.* In vitro* study of released RA is an informative tool for the assessment of the suitability of the base as a carrier for the insertion of liquid lemon balm extract. Scientific studies have shown that RA was accumulating in the epidermis, and the hydrophilic dermis acted as a barrier preventing deeper penetration and entry of RA into systemic blood flow [19]. Substance penetration into or through the skin occurs in a coherent sequence when dissolved molecules released from the dosage form reach the surface of the stratum corneum and penetrate through it [26]. Materials can penetrate through the skin by intracellular, extracellular, and additional “shunt” (through hair follicles and gland ducts) routes [27, 28]. Substance penetration using the intracellular polar route occurs when molecules diffuse through the cytoplasm of dead keratinocytes and the surrounding lipid matrix [29]. For this reason, the human keratinocyte cell line HaCaT was chosen for further studies of the biological effects of the released RA. With these studies, we aimed at determining whether the RA released from the gel has protective effect in normal conditions and under oxidative stress.

### 3.3. Influence of H_2_O_2_ and Rosmarinic Acid on HaCaT Cell Viability during H_2_O_2_ Exposure

**Figure 2:**
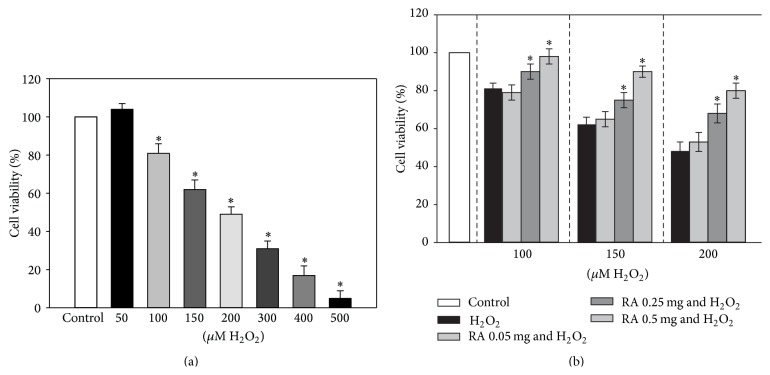
Effect of different concentrations of H_2_O_2_ and RA on HaCaT cell viability. HaCaT cells were treated with (a) different concentrations (50–500 *μ*M) of H_2_O_2_ for 24 hours and (b) different concentrations (0.05–0.5 mg/mL) of RA and H_2_O_2_ (100–200 *μ*M) for 24 hours. Cell viability was assessed using the MTT method. Data are presented as means of the percentage of the untreated control cells ± SE (*n* = 4).

In order to assess the effect of different concentrations of H_2_O_2_ on HaCaT cell viability, the MTT assay was carried out. The results of these experiments are presented in Figure 2(a). EC_50_ was estimated to be 183.7 ± 20.4  *μ*M H_2_O_2_.

For further experiments, we chose RA concentrations (0.05–0.5 mg/mL) according to the results of RA release from the gel received at various time periods and incubated the cells for 24 hours. The results of our experiments revealed (Figure 2(b)) that higher concentrations of RA (0.25–0.5 mg/mL) protected the cells from the damage caused by different concentrations of H_2_O_2_ and enhanced cell viability by 10–24%. Moreover, 0.5 mg/mL of RA restored HaCaT cell viability until the control level in the presence of 100 *μ*M of H_2_O_2_.

It is possible that the protective effect of RA is related to its antioxidant activity. Oxidative stress conditions in cells develop in the presence of an imbalance between ROS generation and endogenous antioxidant defense mechanisms. This state of oxidative stress can affect all important cellular components like proteins, DNA, and membrane lipids, which is considered to be the main mechanism of cell damage [30]. There is research confirming that exogenous molecules from dietary sources such as polyphenols are very efficient in preventing the alteration caused by oxidative stress [31] because they scavenge and suppress the formation of free radicals. Thus the use of preparations with antioxidant activity is critical for cells that are in oxidative stress conditions.

### 3.4. The Antioxidant Activity of Rosmarinic Acid in Normal Conditions and under Oxidative Stress

**Figure 3:**
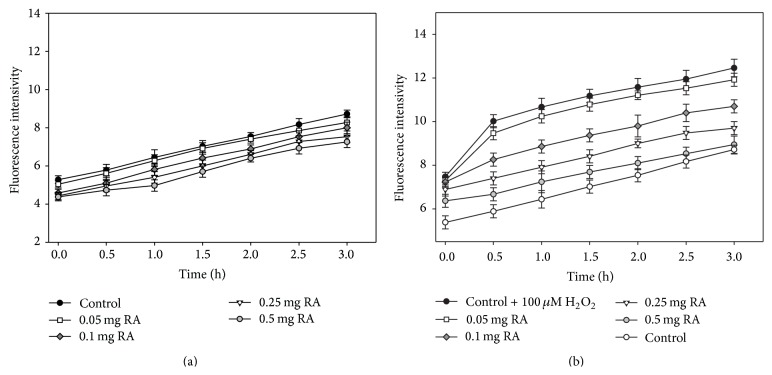
Effects of different concentrations of rosmarinic acid on intracellular ROS generation in HaCaT cells. Cells were preincubated with DCFH-DA (10 *μ*M) for 30 min and then washed twice and (a) treated with different concentrations of RA and (b) treated with 100 *μ*M H_2_O_2_ and different concentrations of RA. Control cells were treated with the same amounts of the solvent. The number of experiments is 3.

In order to evaluate the antioxidant activity of different concentrations of RA in normal conditions and under oxidative stress, in the next series of experiments, we measured the quantity of the intracellular ROS in cells using the DCFH-DA dye (Figures 3(a) and 3(b)). For these experiments, we chose H_2_O_2_ concentration of 100 *μ*M because this is the lowest concentration which after 24 hours statically significantly reduces cell viability. It is known that H_2_O_2_ should relatively easily diffuse into/out of the cell through lipid bilayer or aquaporins. In our experiments (Figure 3(b)) this was clearly visible, since the amount of intracellular ROS in cells that were affected by 100 *μ*M of H_2_O_2_ during the original period (0.5 h) increased by 30%, whereas in control cells (without H_2_O_2_), the amount of ROS increased by an average of 7–10%. The lowest amount of RA (0.05 mg/mL) that affected keratinocyte cells only slightly reduced the amount of ROS (by 4.3%), but all the higher amounts of RA that were used in the study in all the studied time periods significantly reduced intracellular ROS quantity. After 3 hours, the amount of ROS was below 14–28% at 0.1–0.5 mg/mL of RA. In cells affected by 0.5 mg of RA, the amount of ROS was close to that observed in the control cells.

The skin is the interface between the body and its environment and acts as a barrier protecting from adverse external factors. Keratinocytes are the principal cell type comprising the epidermis and constituting 90% of the total amount of epidermal cells [32]. For this reason, the human keratinocyte cell line HaCaT that was used in this research was an excellent model to evaluate the biological effect of the active substance of the modeled preparations. Chemically, rosmarinic acid is a derivative of two connected phenolic acids (coffee and 3,4-dihydroxyphenyl lactic acid) and has 4 OH groups. There are studies showing that it is the most potent antioxidant among the hydroxycinnamic acids [33]. Studies have shown that rosmarinic acid is an effective scavenger of the DPPH radical, and it also scavenges the reactive nitrogen species, peroxynitrite, and various ROS [34]. The results of experiments done with cell cultures show that, through its antioxidant activity, RA could be able to attenuate H_2_O_2_-induced cell injury [35]. The results of our experiments on skin cells also demonstrated that RA had dose-dependent antioxidant activity. In normal conditions, 0.5 mg/mL of RA after 3 hours reduced the amount of intracellular ROS to 23% (Figure 3(a)). Therefore, in normal conditions, the examined preparations can provide protective and antiaging effects. Scientific data confirmed that many external factors (such as UV light, traumas, ultrasound, infections, air pollutants, and cigarette smoke) and internal factors (drugs, contaminated food, and some diseases such as atopic dermatitis) cause increased ROS levels in skin cells. Most of the ROS have a short life span and are immobile (e.g., hydroxyl or superoxide radicals), and they immediately (at the source) react with the surrounding biomolecules. H_2_O_2_ is stable and easily diffusible in an aqueous environment. It easily passes through biological membranes and therefore is suitable to create oxidative stress in experimental conditions. Cell viability-decreasing H_2_O_2_ concentrations determined in our experiments with HaCaT cells (EC_50_  183.7 ± 20.4) were similar to those found by Liu et al. [22]. Investigations of other authors demonstrate that the first signs of cell impairment are noted at 50 *μ*M H_2_O_2_. On the other hand, 150 *μ*M of H_2_O_2_ and 300 *μ*M of H_2_O_2_ are relatively high doses to induce apoptosis in Jurkat T cells and in HaCaT cells, respectively [32, 36]. It was shown that very severe oxidative stress activates a large number of signaling pathways in cells and causes cell death via either apoptotic or necrotic mechanisms [37]. The RA concentration of 0.05 mg/mL slightly reduced the amount of intracellular ROS and had only limited effect on the oxidative state of cells. Higher amounts of RA (0.1 and 0.25 mg/mL) decreased the level of ROS by 14 and 22%, respectively. However, the amount of radicals in cells was still higher than that in the controls. In normal conditions, skin cells do not sustain that much damage from harmful environmental factors to initiate the production of such large amounts of ROS that they would cause cell death. Only with 0.5 mg/mL of RA, a decrease in the amount of ROS by 28% was achieved, and the remaining amount of ROS was similar to that in the control cells. Based on these findings, it is clear that preparations with lemon balm extract can reduce the amount of ROS increased because of unfavorable external conditions, the effect of internal factors on the body, or a pathologic skin process in intracellular keratinocytes.

## 4. Conclusions

Lemon balm hydrogels were modeled using carbomer, methylcellulose, and mixtures of them as gelling materials. The study showed that the used gelling material affected the physicochemical properties of semisolid preparations. The results of biopharmaceutical tests* in vitro* showed that even though the highest amount of released RA was found in the methylcellulose-based gels, carbomer (up to 1.0%), due to its semisolid consistency, was a more appropriate base for the insertion of lemon balm extracts. The results of biological investigations showed that rosmarinic acid, depending on the concentration, reduced the amount of intracellular ROS in human keratinocyte cells and enhanced cell viability under oxidative stress.

## Figures and Tables

**Table 1:** Composition of experimental lemon balm hydrogels.

Composition (%)	Gels								
	N1	N2	N3	N4	N5	N6	N7	N8	N9
Carbomer 980	0.5	1.0	1.5	0.5	0.5	0.5	—	—	—
Methylcellulose 15cP	—	—	—	1.0	2.0	4.0	1.0	2.0	4.0
Propylene glycol	20.0	20.0	20.0	20.0	20.0	20.0	20.0	20.0	20.0
1.0% NaOH	qs ad pH 7	qs ad pH 7	qs ad pH 7	qs ad pH 7	qs ad pH 7	qs ad pH 7	—	—	—
*Melissa officinalis* (L.) dry extract	4.0	4.0	4.0	4.0	4.0	4.0	4.0	4.0	4.0
Purified	qs ad 100.0	qs ad 100.0	qs ad 100.0	qs ad 100.0	qs ad 100.0	qs ad 100.0	qs ad 100.0	qs ad 100.0	qs ad 100.0

**Table 2:** Results of quality control testing in gels.

Quality parameter	Gels								
	N1	N2	N3	N4	N5	N6	N7	N8	N9
pH value	7.45 ± 0.22	7.62 ± 0.31	7.75 ± 0.18	7.35 ± 0.21	7.20 ± 0.19	7.08 ± 0.13	5.84 ± 0.15	6.14 ± 0.27	6.32 ± 0.30
Dynamic viscosity, Pa s	2.45 ± 0.12	3.51 ± 0.08	3.86 ± 0.10	2.64 ± 0.14	2.90 ± 0.08	2.98 ± 0.03	0.01 ± 0.01	0.07 ± 0.01	0.61 ± 0.08
